# Identification of quantitative trait loci for survival in the mutant dynactin p150Glued mouse model of motor neuron disease

**DOI:** 10.1371/journal.pone.0274615

**Published:** 2022-09-15

**Authors:** Guillermo M. Alexander, Terry D. Heiman-Patterson, Frank Bearoff, Roger B. Sher, Laura Hennessy, Shannon Terek, Nicole Caccavo, Gregory A. Cox, Vivek M. Philip, Elizabeth A. Blankenhorn

**Affiliations:** 1 Temple MDA/ALS Center of Hope, Philadelphia, Pennsylvania, United States of America; 2 Department of Neurology, Lewis Katz School of Medicine of Temple University, Philadelphia, Pennsylvania, United States of America; 3 Department of Microbiology Drexel University College of Medicine, Philadelphia, Pennsylvania, United States of America; 4 Department of Neurobiology and Behavior, Stony Brook University, Stony Brook, New York, United States of America; 5 The Children’s Hospital of Philadelphia, Philadelphia, Pennsylvania, United States of America; 6 The Jackson Laboratory, Bar Harbor, Maine, United States of America; First Affiliated Hospital of Dalian Medical University, CHINA

## Abstract

Amyotrophic lateral sclerosis (ALS) is the most common degenerative motor neuron disorder. Although most cases of ALS are sporadic, 5–10% of cases are familial, with mutations associated with over 40 genes. There is variation of ALS symptoms within families carrying the same mutation; the disease may develop in one sibling and not in another despite the presence of the mutation in both. Although the cause of this phenotypic variation is unknown, it is likely related to genetic modifiers of disease expression. The identification of ALS causing genes has led to the development of transgenic mouse models of motor neuron disease. Similar to families with familial ALS, there are background-dependent differences in disease phenotype in transgenic mouse models of ALS suggesting that, as in human ALS, differences in phenotype may be ascribed to genetic modifiers. These genetic modifiers may not cause ALS rather their expression either exacerbates or ameliorates the effect of the mutant ALS causing genes. We have reported that in both the G93A-hSOD1 and G59S-hDCTN1 mouse models, SJL mice demonstrated a more severe phenotype than C57BL6 mice. From reciprocal intercrosses between G93A-hSOD1 transgenic mice on SJL and C57BL6 strains, we identified a major quantitative trait locus (QTL) on mouse chromosome 17 that results in a significant shift in lifespan. In this study we generated reciprocal intercrosses between transgenic G59S-hDCTN1 mice on SJL and C57BL6 strains and identified survival QTLs on mouse chromosomes 17 and 18. The chromosome 17 survival QTL on G93A-hSOD1 and G59S-hDCTN1 mice partly overlap, suggesting that the genetic modifiers located in this region may be shared by these two ALS models despite the fact that motor neuron degeneration is caused by mutations in different proteins. The overlapping region contains eighty-seven genes with non-synonymous variations predicted to be deleterious and/or damaging. Two genes in this segment, NOTCH3 and Safb/SAFB1, have been associated with motor neuron disease. The identification of genetic modifiers of motor neuron disease, especially those modifiers that are shared by SOD1 and dynactin-1 transgenic mice, may result in the identification of novel targets for therapies that can alter the course of this devastating illness.

## Introduction

Amyotrophic lateral sclerosis (ALS) is a degenerative motor neuron disease (MND) resulting in progressive paralysis, muscle atrophy, and ultimately death, with a median survival of less than five years. However, there is variability in ALS severity, with 20% of patients living longer than five years and 10% of patients living 10 years or more. Although most cases of ALS are sporadic (SALS), approximately 5–10% of ALS cases are familial (FALS), with mutations associated with over 40 genes [1–4]. There is also wide variation of ALS symptoms even within families carrying the same familial mutation; the disease may develop in one sibling and not in another despite the presence of the same mutation in both. Although the cause of this phenotypic variation has not been determined, it is likely related to genetic modifiers of disease expression inherent in the genetic heterogeneity even within members of the same family.

Mutations in the Cu/Zn superoxide dismutase (SOD1) gene were the first identified in FALS [5, 6]. The identification of SOD1 mutations led to the development of a transgenic mouse model of ALS carrying the human G93A mutant allele [7]. These mice develop motor neuron pathology and clinical symptoms remarkably similar to those seen in ALS patients [8–11]. Similar to families with SOD1 mutations, there are background-dependent differences in disease phenotype in transgenic G93A-hSOD1 mice. We have previously reported that the G93A-hSOD1 transgene in ALR/LtJ, NOD.Rag1KO, SJL/J or C3H/HeJ backgrounds show a more severe phenotype whereas a milder phenotype is observed in C57BL6 (B6), C57BL/10ScSnJ (B10), BALB/cByJ and DBA/2J inbred strains [12, 13]. Utilizing these mouse strains, we identified a major quantitative trait locus (QTL) on mouse chromosome (Chr) 17 that results in a significant shift in lifespan [14], suggesting that, as in human ALS, the differences in phenotype can be ascribed to genetic modifiers of motor neuron disease.

The identification of other FALS associated mutations has led to additional animal models of motor neuron disease such as the mutant (G59S) human dynactin p150Glued transgenic mouse (G59S-hDCTN1). This model is based on a slowly progressive, autosomal dominant lower motor neuron variant of FALS in humans that is linked to a mutation in the p150Glued subunit of the dynactin complex [15]. Transgenic mice that carry the mutated human dynactin-1 transgene (G59S-hDCTN1) demonstrate pathologic changes and clinical features seen in SALS [16, 17]. These mice develop spontaneous tremors between five and six months of age. Once near end-stage of the disease, the mice start to lose weight, stop grooming, and eventually become paralyzed [16]. Similar to our finding in G93A-hSOD1 mice [12, 13], we observed a more severe phenotype with significant acceleration of disease onset and age of death in G59S-hDCTN1 mice bred onto the SJL background and a milder phenotype with delayed onset and extended lifespan when bred onto the B6 background [18].

In this study, we report the results of QTL analysis of an intercross between G59S-hDCTN1 transgenic mice on a resistant (C57BL/6J) and a susceptible (SJL/J) mouse strain.

## Materials and methods

### Animals

Mice from two inbred strains were used in this study: C57BL/6J (B6) and SJL/J (SJL). G59S-hDCTN1 mice were originally produced by Dr. Philip C. Wong at The Johns Hopkins University School of Medicine by injecting the transgene containing the G59S substitution into fertilized eggs from C57BL6/SJL F1 hybrid mice [17]. The mice were then maintained on a mixed B6xSJL/F1 background. From these mice, we developed two inbred strains (C57BL/6J and SJL/J) by backcrossing the original mixed B6/SJL mice to either pure B6 or SJL for seven generations [18]. An intercross was generated from short-(SJL), and long-(B6) lived strains for mapping of quantitative trait loci that modify the onset and survival of G59S-hDCTN1 transgenic mice. The F1 mice were derived from crosses in both directions [SJL ♀ x B6 ♂] and [B6 ♀ x SJL ♂] and in all but one litter, the transgene was carried in the male. From the F1 cross, 167 F2 mice were generated. The F2 generation is the first generation where both parental phenotypes occur for an autosomal gene. In an autosomal gene there are four possible genotypes B6xB6 (BB), B6xSJL or SJLxB6 (BS) and SJLxSJL (SS). Thus, in an F2 cross at any location of an autosomal gene, the genotype is such that at that location 25% of the mice are BB, 50% BS and 25% SS. This is not the case for the sex chromosomes. One of the X chromosomes in all females but not males carry the paternal grandmother (pgm) X chromosome (either all S or all B). At the other X chromosome, any region can be either B or S depending on cross over events. In males, the Y chromosome is always that of the paternal grandfather (either all S or all B).

The mice were maintained in humidity- and temperature-controlled rooms and fed PicoLab rodent diet 20 ad libitum with free access to water. The mice were genotyped using DNA isolated from a 0.5 cm piece of mouse tail. Isolation was performed with the Qiagen DNeasy Blood & Tissue Kit (Qiagen, Germantown, MD 20874). All animal studies were approved by the Institutional Animal Care and Use Committee (IACUC) of Temple University and were carried out in accordance with the National Institute of Health guide for the care and use of laboratory animals.

### Genotyping

The presence of the G59S-hDCTN1 transgene was determined by PCR as previously described [17]. When breeding mice each carrying one copy of the G59S-hDCTN1 transgene, the transgene copy number of the offspring was determined by quantitative real-time PCR using mouse IL-2 as a reference gene as previously described [19]. Assays were performed, in duplicate, on a Chromo 4 Quantitative PCR System (Bio-Rad, Hercules, CA). For the QTL analysis, the F2 mice were genotyped with 136 SNPs across the genome that differentiate C57BL/6J from SJL/J mice (S1 Table).

### Life span

Life span was defined as age of sacrifice for any reason. Mice were sacrificed when they demonstrated limb paralysis or were unable to right themselves in 10 seconds when placed on their side. In addition, mice were also euthanized if their health deteriorated to a degree such that the veterinary staff recommended euthanasia. We differentiated between these two phenotypes. Euthanasia due to limb paralysis or inability to right themselves was defined as “survival”, whereas euthanasia due to health deterioration was defined as “health sacrifice” (health_sac). Euthanasia was performed by an overdose of CO_2_ followed by cervical dislocation. All efforts were taken to minimize pain and discomfort. Progressive deterioration of the animals’ health leading to death was not allowed. Natural death was not an end point in this study.

### QTL analysis

QTL analysis was performed using R/QTL version 1.46.2 [20] running in R for Windows version 4.0.5. A book by Broman and Sen provides a review of statistical QTL mapping in experimental crosses and is an excellent guide to the use of the R/qtl software package [21]. For one-dimensional scans, pseudo-markers were generated at 2 centimorgan (cM) spacing for each Chr using the Carter–Falconer map function and whole genome scans were performed using 128 imputations [22]. One thousand permutations were performed to determine the thresholds for QTL detection [23]. Logarithm of the odds (LOD) scores were calculated with no covariates and with the inclusion of sex and the paternal grandmother (pgm) of the F2 cross as both additive and interactive covariates. Four thresholds 1%, 5%, 10% and 63% were calculated from the permutation results. QTL with LOD score above the 1% threshold were considered significant, while those above the 63% threshold were considered suggestive [24].

For two-dimensional scans, pair wise scans were performed using 2 cM spacing. All possible pairs of QTL locations on each Chr were tested for association with the life span. The likelihood from the full model (pseudo-marker pair and the interaction between them) and the null model (no genetic effect) was compared and LOD scores were calculated.

QTL and possible QTL*QTL interactions identified from single QTL scan and pair wise scan were fit into multiple regression models. By doing so, variations of the phenotype in the models were estimated. P values for terms in the multiple regression model were calculated. Terms were dropped sequentially until all the terms in the model were significant at 1% level (p<0.01) for main QTL effects and 0.1% (p<0.001) for the interaction effects. The position of the QTL was refined using the refine QTL command. This routine iteratively scans the QTL to identify the positions with the maximum LOD score.

### Genomic variations

The SJL sequence was obtained in BAM format from the Sanger Institute web site (https://www.sanger.ac.uk/data/mouse-genomes-project/). Sequence variation (SNPs, insertions and deletions) were determined in the QTL regions with samtools and bcftools [25] using the genome sequence file for SJL and the C57BL6 reference genome as inputs. Variations in the coding regions were determined using PROVEAN [26] (http://provean.jcvi.org/index.php). PROVEAN was also used to determine variations that resulted in non-synonymous protein changes as well as changes predicted to be deleterious and/or damaging.

### Statistics

Significance between groups was determined by analysis of variance (ANOVA) using the Tukey-Kramer post-hoc multiple comparison test. The data was considered significantly different if p< 0.05 unless otherwise noted. Statistical calculations were accomplished with the aid of SYSTAT version 13 (SYSTAT Software Inc., Chicago, IL).

## Results

### Effect of genetic background on lifespan

We have previously shown that G59S-hDCTN1 mice demonstrate a shortened lifespan when bred on a SJL background (273.3 ± 7.1 days, N = 41) as compared to mice on the B6 background (444.4 ± 4.6 days, N = 49) [18]. There was no significant difference in lifespan (p>0.05) between male and female mice in either background [18]. The mean lifespan ± standard error in the 167 mice in the B6xSJL F2 cross in this study was 492.8 ± 9.4 days. The lifespan of the G59S-hDCTN1 transgenic mice in the F2 cross was significantly different (p<0.05) from transgenic mice in either the B6 or the SJL background. The F2 B6xSJL mice were classified into two groups based on the reason for euthanasia. The first group of 125 mice were euthanized due to limb paralysis or failure to right themselves when placed on their side. This group was named “survival” and demonstrated a mean age at sacrifice of 499.9 ± 10.6 days. The second group of 42 mice were euthanized due to causes other than limb paralysis or failure to right themselves such as; enlarged abdomens, liver tumors, prolapsed rectum loss of greater than 30% of their peak body weight and eye ulcers. This group was named “health_sac” and demonstrated a mean age at sacrifice of 471.5 ± 19.8 days. There was no statistically significant difference in the age at sacrifice (p>0.05) between groups.

### Distribution of the survival and health_sac phenotypes of the F2 mice

**Fig 1:**
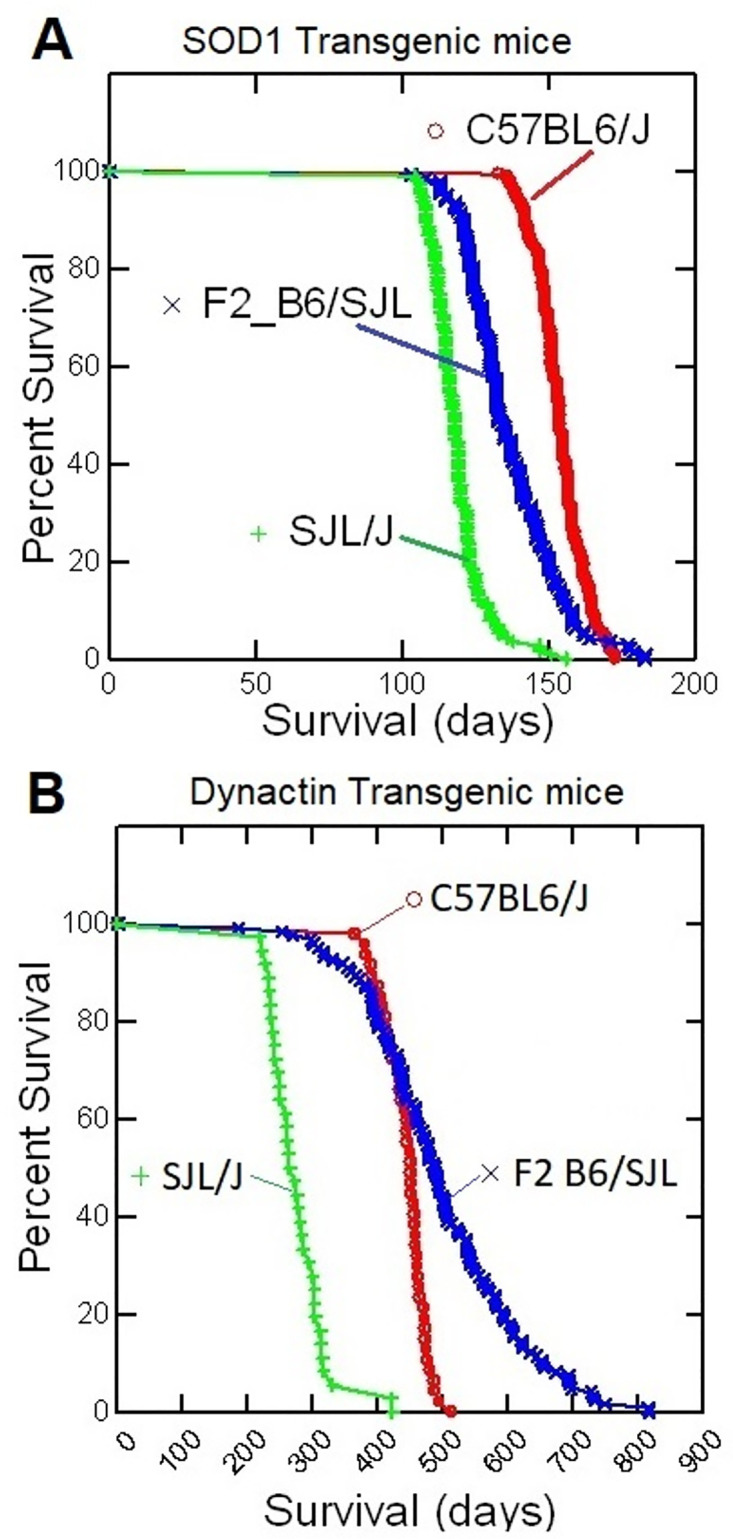
Survival curves for SOD1 and dynactin-1 transgenic mice. Survival curves for transgenic C57BL6/J, SJL/J mice and their F2 cross with [A] the G93A human SOD1 transgene or [B] the G59S human dynactin-1 transgene.

The survival phenotype distribution of the F2 mice in this study was very different from the survival phenotype of the G93A-hSOD1 F2 mice in our previous linkage study [14]. The survival distribution of the G93A-hSOD1 F2 was intermediate between the B6 and SJL distributions (Fig 1A). In contrast, 40% of the F2 mice in this study demonstrated greater survival than the longest-lived mouse in the B6 group (Fig 1B). This distribution also applied to the health_sac phenotype, where 38% of the mice lived longer than the longest-lived mouse in the B6 group, suggesting that combinations of B6 and SJL alleles result in phenotypes milder than the phenotype observed in B6 mice.

### Effect of sex and F2 paternal grandmother on lifespan

There was a small but not significant (p = 0.1621) difference in the life span of the F2 mice by sex, however, male mice that required euthanasia due to limb paralysis or inability to right themselves (survival) lived significantly longer (p = 0.0315) than females. There was no difference in the time of sacrifice of the health_sac group by sex (Table 1).

**Table 1:** Effect of sex on life span of F2 G59S-hDCTN1 mice.

Phenotype	Males		Female		t-Test
	N	Mean±se	N	Mean±se	p value
Life span	75	507.3±15.5	92	480.9±11.3	0.1621
Survival	49	528.1±17.7	76	481.7±12.8	0.0315
Health_sac	26	468.0±28.8	16	477.1±23.7	0.8266

All groups demonstrate a significant difference in life span depending on the direction of the F2 cross. Mice with the paternal grandmother (pgm) of the F2 cross in the B6 strain lived significantly longer (p<0.05) than mice with pgm in the SJL strain (Table 2).

**Table 2:** Effect of the paternal grandmother of the F2 cross on life span of G59S-hDCTN1 mice.

Phenotype	C57BL6/J pgm		SJL/J pgm		t-Test
	N	Mean±se	N	Mean±se	p value
Life span	99	525.2±12.6	68	445.6±11.8	0.00002
Survival	67	536.2±16.1	58	458.0±10.9	0.00016
Health_sac	32	502.1±19.2	10	373.7±44.9	0.00432

### QTL analysis identifies regions that modify the survival and health_sac phenotypes

**Fig 2:**
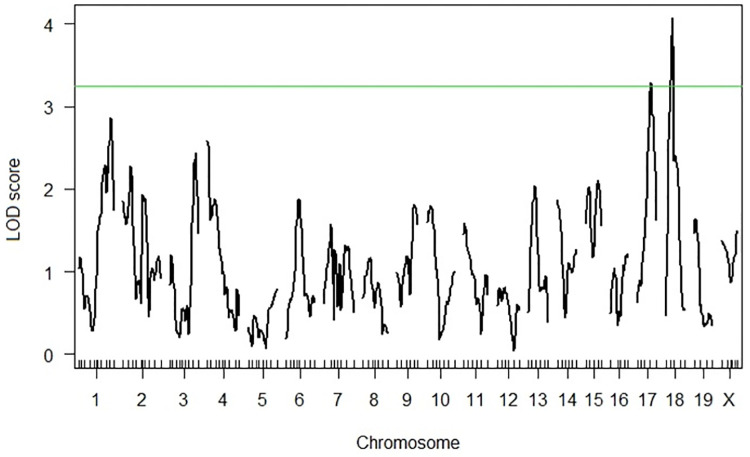
Whole genome LOD plot for the survival phenotype of the G59S-hDCTN1 mice. LOD plot of a whole genome scan for the Survival phenotype with pgm as an additive covariate and sex as an interactive covariate. Chromosome locations are on the X axis, LOD score on the Y axis. The green line indicates suggestive QTL threshold at a LOD of 3.2.

Linkage analysis revealed suggestive QTLs on Chrs 17 and 18 for the survival phenotype (Fig 2, S2 Table). In addition, two dimensional scans reveal suggestive interactions between the Chr 17 QTL and a region on Chr 4 as well as interactions between the X Chr and regions on Chrs 5, 9, 13 and 18 (S4 Table).

**Fig 3:**
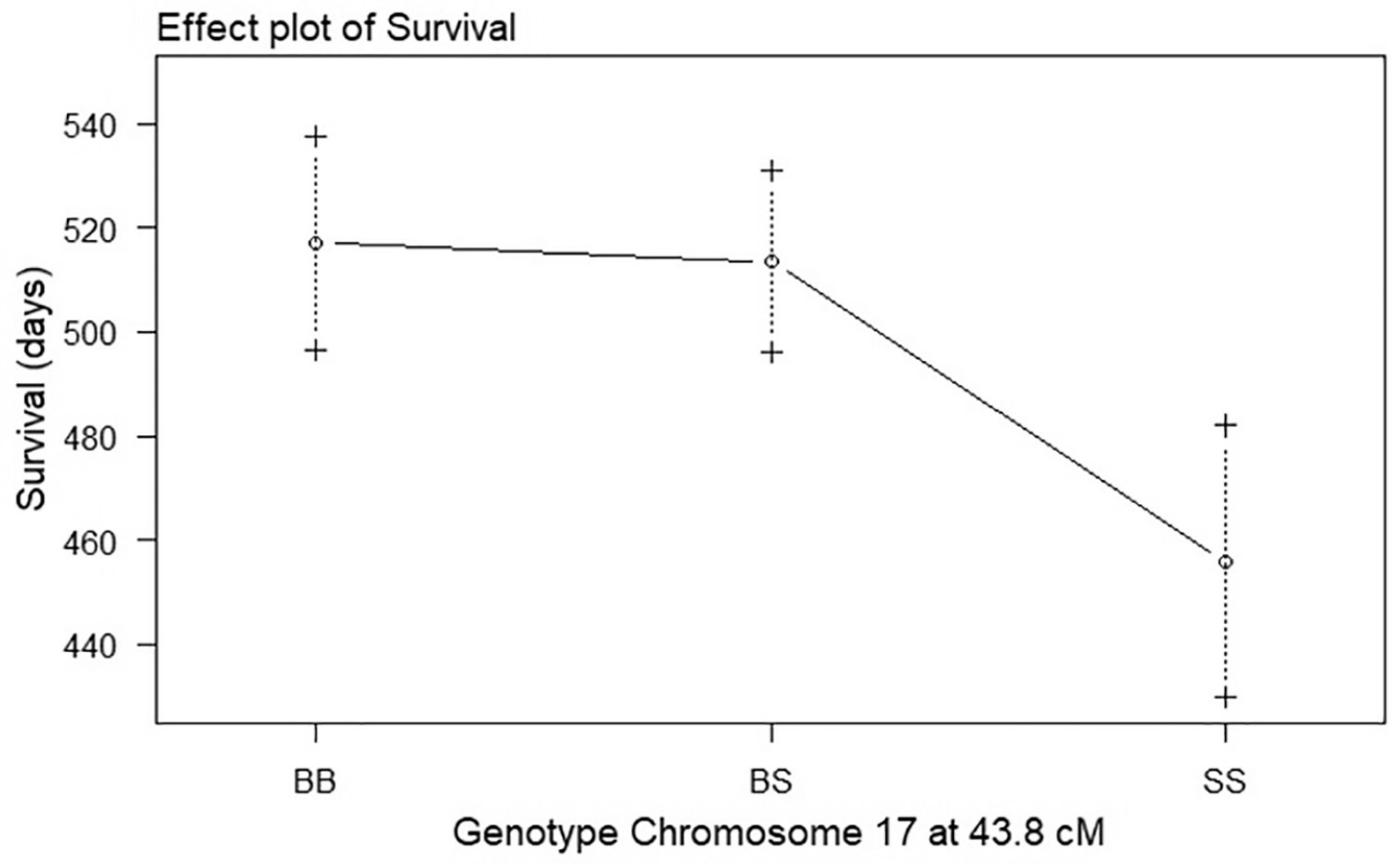
Effect plot of genotype on survival at the chromosome 17 QTL of G59S-hDCTN1 mice. Effect of the genotype at chromosome 17, 43.8 cM on survival. The survival in days is plotted as the mean ± SE. The genotypes are BB for homozygous B6, SS for homozygous SJL and BS for heterozygous B6/SJL.

**Fig 4:**
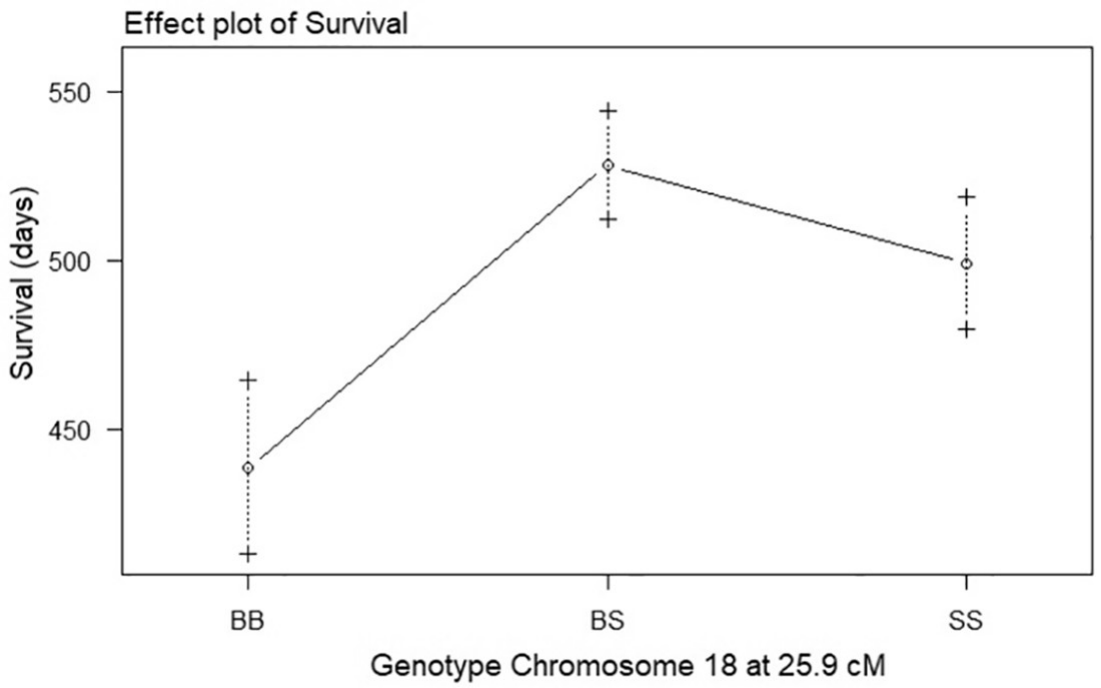
Effect plot of genotype on survival at the chromosome 18 QTL of G59S-hDCTN1 mice. Effect of genotype at chromosome 18, 25.9 cM on survival. The survival in days is plotted as the mean ± SE. The genotypes are BB for homozygous B6, SS for homozygous SJL and BS for heterozygous B6/SJL.

All putative QTLs identified by the one-dimensional scans as well as interactions between QTLs identified by the two-dimensional scans were fitted into a multiple regression model using the function *fitqtl*. The QTL locations were then refined using the function *refineqtl*. As stated in the methods, the multiple regression model require significance at the 1% level (p<0.01) for main QTL effects and a more stringent 0.1% (p<0.001) for interaction effects. For the survival phenotype, both the Chr 17 and 18 QTLs were significant at the 1% level (p<0.01). Following running *refineqtl* the maximum likelihood position for the Chr 17 QTL remained at 43.8 cM whereas the Chr 18 QTL was calculated to be at 25.9 cM. The effects of the QTLs differed; the BB genotype at the Chr17 QTL demonstrated longer survival than the SS (Fig 3), whereas at the Chr18 QTL the BB genotype demonstrated shorter survival than the SS (Fig 4). None of the QTL*QTL interactions identified in the two-dimensional scans were significant at the 0.1% level. The refined QTL locations and QTL intervals for the Chrs 17 and 18 QTLs of the survival phenotype are shown in Table 3.

**Table 3:** Multiple regression model for the survival phenotype in G59S-hDCTN1 mice.

Source	Additive covar	Interactive covar	Location (cM)	Interval (cM)	Percent variance	F value	p value
Chr 17	Sex		43.8	27.8–57.8	8.651	3.596	0.00840
Chr 17		Sex	43.8	27.8–57.8	6.303	5.241	0.00662
Chr 18	Pgm		25.9	13.9–37.9	6.235	5.184	0.00697
Pgm					10.397	17.29	6.18x10^-5^
Sex					8.435	4.636	0.00404

Linkage analysis also revealed suggestive QTLs on Chrs 4, 6, 10, 13 and 15 for the health_sac phenotype (S3 Table) and two-dimensional scans also revealed interactions for the health_sac phenotype between regions on Chrs 4 and 13, Chrs 5 and 10, Chr 6 and 19 as well as Chrs 13 and 19 (S4 Table).

For the health_sac phenotype, when fitted into multiple regression models, the QTLs on Chrs 6 and 15 were significant at the 1% level (p<0.01). Following running *refineqtl* the maximum likelihood position for the Chr 15 health_sac QTL remained at 16.3 cM whereas the Chr 6 QTL was calculated to be at 33.5 cM. The effects of the Chr 6 and 15 QTLs differed. Mice with the BB genotype at Chr 6 demonstrated longer life span due to health sacrifice than mice with the SS genotype, whereas at the Chr 15 QTL mice with the BS genotype demonstrated longer life span due to health sacrifice than mice with either the BB or SS genotype, (S1 and S2 Figs). None of the QTL*QTL interactions identified in the two-dimensional scans were significant at the 0.1% level. The refined QTL locations and QTL intervals for the Chrs 6 and 15 QTLs of the health_sac phenotype is shown in (Table 4).

**Table 4:** Multiple regression model for the health sac phenotype in G59S-hDCTN1 mice.

Source	Additive covar	Interactive covar	Location (cM)	Interval (cM)	Percent variance	F value	p value
Chr 6		Sex	33.5	1.5–61.5	14.89	6.861	0.00341
Chr 15	Pgm		16.3	10.3–50.0	18.38	4.234	0.00753
Chr 15		Pgm	16.3	10.3–50.0	14.78	6.808	0.00354
Pgm					30.76	9.446	1.38x10^-4^
Sex					18.64	5.724	0.00308

## Discussion

We have reported a more severe phenotype with significant acceleration of disease onset and decreased life span in G59S-hDCTN1 mice bred onto the SJL background and a milder phenotype with delayed onset and increased life span when bred onto the B6 background [18]. The present study genetically mapped changes in life span through genome-wide analysis of an intercross between G59S-hDCTN1 transgenic mice on the mild phenotype (C57BL/6J) and the severe phenotype (SJL/J) mouse strains. The result of this study demonstrates that the causes of decreased life span in the G59S-hDCTN1 transgenic mice are complex. In contrast to the high copy G93A-hSOD1 transgenic mice, where euthanasia is precipitated by hind limb paralysis or inability to right itself, deteriorating health requiring euthanasia in the G59S-hDCTN1 transgenic mice resulted from multiple conditions. We split lifespan into two phenotypes: euthanasia due to limb paralysis or inability to right themselves as “survival”, whereas euthanasia due deteriorating health such that the veterinary staff recommended euthanasia as “health_sac”.

The effect of sex on lifespan differed depending on the phenotype. Male mice in the survival phenotype lived significantly longer (p = 0.0315) than females. This was not the case for the health_sac phenotype. However, both phenotypes demonstrated a significant difference in life span depending on the strain of the paternal grandmother (pgm). Mice with pgm in the SJL strain had reduced life span (p<0.01) compared with mice with pgm in the B6 strain. Suggesting that genetic elements in the X chromosome that differ between strains modify phenotype, either directly, or in combination with autosomal genes.

This study identified QTL loci on mouse Chrs 17 and 18 for the survival phenotype and on Chrs 6 and 15 for the health sacrifice phenotype that when fitted into multiple regression models were significant at the 1% level (p<0.01). The two phenotypes clearly differed. The survival phenotype demonstrated a significant (p<0.05) difference with sex whereas the health_sac phenotype did not. In addition, the genetic loci identified for each phenotype were located on different Chrs suggesting that different genetic elements modify the survival and health sacrifice phenotypes in the G59S-hDCTN1 mouse. Given that the principal aim of our study is to identify genetic modifiers of disease expression in models of motor neuron disease, we focused the discussion on the survival phenotype.

Our previous linkage study on G93A-hSOD1 transgenic mice identified QTLs on Chrs 17 and 7 for the survival phenotype. At both QTL locations, mice with BB alleles demonstrated longer survival than mice with SS alleles. The allele order for the survival phenotype at the QTL locations was BB>BS>SS. In this study, the QTL on chromosome 17 followed a slightly different pattern, with an allele order for survival of BB = BS>SS (Fig 3). In addition, the QTL on Chr 18, demonstrates a completely different pattern with an allele order for survival of BS>SS>BB (Fig 4). In other words, mice that are heterozygous or homozygous for SJL alleles at the Chr 18 QTL location demonstrated longer survival than mice with B6 alleles. On average, autosomal chromosome locations in the F2 mice are 25% BB, 50% BS and 25% SS. The survival of mice with a BS genotype at the Chr 17 QTL is no different than mice with a BB genotype, whereas BS mice at the Chr 18 QTL have longer survival than both SS and BB. The fact that half of the F2 mice have BS genotype at the QTL locations may explain why many B6/SJL F2 mice demonstrate longer survival than mice on a pure B6 background (Fig 1B).

**Fig 5:**
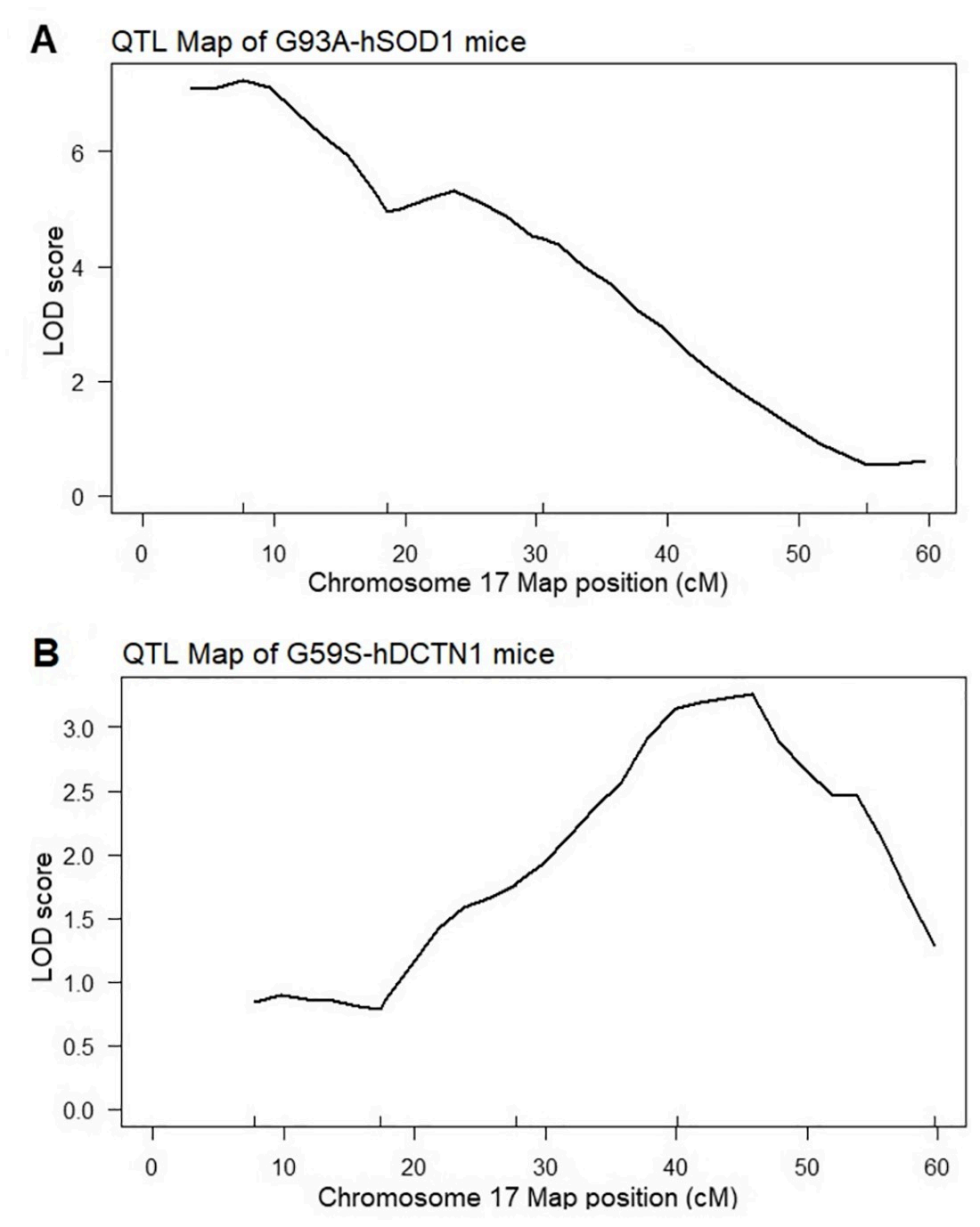
LOD plots of chromosome 17 QTLs of SOD1 and dynactin-1 transgenic mice. Chromosome 17 LOD plots. [A] Chromosome 17 Survival QTL in F2 B6xSJL G93A-hSOD1 mice. [B] Chromosome 17 Survival QTL in F2 B6xSJL G59S-hDCTN1 mice.

The location of the Chr 17 survival QTL on the G93A-hSOD1 mouse partly overlaps with the Chr 17 survival QTL in the G59S-hDCTN1 mouse (Fig 5). The survival QTL in SOD1 transgenic mice demonstrates the highest LOD scores between 3 and 17 cM (Fig 5A). In contrast, the survival QTL in dynactin-1 transgenic mice demonstrate the highest LOD scores between 28 and 58 cM (Fig 5B). However, there is an area between 17 and 40 cM were the QTL maps overlap (Fig 5), suggesting that genetic elements in this region may modify survival in both models of motor neuron disease.

The QTL region on Chr 17 contains 251 genes where SJL mice demonstrate non-synonymous variations (S5 Table). Ninety-six of those genes have PROVEAN or SIFT scores that predict the variations to be deleterious and/or damaging to protein function (Table 5).

**Table 5:** Protein coding genes in the Chr17 QTL of G59S-hDCTN1 mice demonstrating non-synonymous variations deemed to be deleterious and/or damaging.

Mouse Gene Symbol	Mouse Gene ID	Human Gene Symbol	Mouse Gene Name	Chr 17 Location GRCm39	Del/Dam # Vars
Rrp1b	72462	RRP1B	ribosomal RNA processing 1B	32255074–32281839	2
Notch3	18131	NOTCH3	notch 3	32339794–32385826	1
Zfp799	240064	ZNF799	zinc finger protein 799	33034423–33049235	1
Cyp4f13	170716	CYP4F3	cytochrome P450, family 4, subfamily f, polypeptide 13	33143662–33166376	1
Phf8-ps	74042		PHD finger protein 8, pseudogene	33283117–33286999	2
Morc2b	240069	MORC2	microrchidia 2B	33332325–33358657	1
Zfp955a	77652		zinc finger protein 955A	33458692–33474119	1
Zfp955b	100043468		zinc finger protein 955B	33508518–33526215	2
Marchf2	224703	MARCHF2	membrane associated ring-CH-type finger 2	33904666–33937644	2
Ndufa7	66416	NDUFA7	NADH: ubiquinone oxidoreductase subunit A7	34043546–34057291	1
Vps52	224705	VPS52	VPS52 GARP complex subunit	34174786–34186009	10
H2-K1	14972	HLA-A	histocompatibility 2, K1, K region	34214991–34219321	24
Rxrb	20182	RXRB	retinoid X receptor beta	34250786–34257373	1
Tap1	21354	TAP1	transporter 1, ATP-binding cassette, sub-family B (MDR/TAP)	34406527–34416199	3
Gm15821	100502931		predicted gene 15821	34430286–34433433	1
H2-Ob	15002	HLA-DOB	histocompatibility 2, O region beta locus	34457877–34473388	3
H2-Ab1	14961	HLA-DQB1	histocompatibility 2, class II antigen A, beta 1	34476663–34488393	4
H2-Aa	14960	HLA-DQA1	histocompatibility 2, class II antigen A, alpha	34501718–34506797	3
H2-Eb1	14969	HLA-DRB5	histocompatibility 2, class II antigen E beta	34524841–34535648	3
H2-Eb2	381091		histocompatibility 2, class II antigen E beta2	34544639–34560386	1
BC051142	407788		cDNA sequence BC051142	34617794–34679708	4
Btnl4	632126		butyrophilin-like 4	34685536–34696292	11
Btnl6	624681		butyrophilin-like 6	34726778–34736326	9
Notch4	18132	NOTCH4	notch 4	34783242–34807477	1
Tnxb	81877	TNXB	tenascin XB	34879431–34938789	4
Nelfe	27632	NELFE	negative elongation factor complex member E, Rdbp	35069367–35075348	3
Ehmt2	110147	EHMT2	euchromatic histone lysine N-methyltransferase 2	35117445–35133028	1
D17H6S56E-5	110956	D6S56E 5	DNA segment, Chr 17, human D6S56E 5	35215654–35219722	1
Ly6g6f	433099	LY6G6F	lymphocyte antigen 6 complex, locus G6F	35299456–35304586	1
H2-D1	14964	HLA-A	histocompatibility 2, D region locus 1	35481706–35486475	20
H2-Q1	15006	HLA-A	histocompatibility 2, Q region locus 1	35539381–35544075	2
H2-Q2	15013	HLA-A	histocompatibility 2, Q region locus 2	35561218–35565738	25
H2-Q4	15015	HLA-A	histocompatibility 2, Q region locus 4	35598593–35604266	33
H2-Q6	110557	HLA-A	histocompatibility 2, Q region locus 6	35643826–35649031	2
Muc21	672682	MUC21	mucin 21	35928815–35937529	11
Mucl3	268949	MUCL3	mucin like 3	35946644–35954587	2
Vars2	68915	VARS2	valyl-tRNA synthetase 2, mitochondrial	35966526–35978484	2
Gtf2h4	14885	GTF2H4	general transcription factor II H, polypeptide 4	35978622–35984631	1
Mdc1	240087	MDC1	mediator of DNA damage checkpoint 1	36152407–36170562	1
Ppp1r18	76448	PPP1R18	protein phosphatase 1, regulatory subunit 18	36176485–36186488	1
H2-T24	15042		histocompatibility 2, T region locus 24	36316587–36331452	1
H2-T23	15040	HLA-E	histocompatibility 2, T region locus 23	36340665–36343747	11
H2-T22	15039		histocompatibility 2, T region locus 22	36348020–36353639	17
Gm11127	100529082	HLA-F	predicted gene 11127 (H2-K1)	36366708–36369263	3
Gm7030	630294	HLA-F	predicted gene 7030 (H2-t9)	36420611–36440317	1
2410017I17Rik	675325		RIKEN cDNA 2410017I17 gene	36455910–36474068	35
Gm8909	667977	HLA-A	predicted gene 8909 (H2-gs17)	36475335–36479429	21
H2-T3	15043		histocompatibility 2, T region locus 3	36496464–36501179	13
H2-M10.1	14985		histocompatibility 2, M region locus 10.1	36633750–36637047	1
H2-M11	224754		histocompatibility 2, M region locus 11	36857967–36860142	1
H2-M1	224756		histocompatibility 2, M region locus 1	36980900–36983111	1
H2-M10.5	224761		histocompatibility 2, M region locus 10.5	37083802–37087126	1
Polr1has	76416		RNA polymerase I subunit H, antisense	37269484–37276517	3
Olfr90	258469	OR2H2	olfactory receptor 90	37394763–37399391	1
Olfr92	258448		olfactory receptor 92	37421404–37430975	2
Olfr93	258051		olfactory receptor 93	37451309–37472385	1
Adgrf1	77596	ADGRF1	adhesion G protein-coupled receptor F1	43581220–43635628	1
Supt3	109115	SUPT3H	SPT3, SAGA and STAGA complex component	45088039–45430177	3
Aars2	224805	AARS2	alanyl-tRNA synthetase 2, mitochondrial	45817767–45831769	2
Hsp90ab1	15516	HSP90AB1	heat shock protein 90 alpha (cytosolic), class B member 1	45878701–45884197	12
1600014C23Rik	72240		RIKEN cDNA 1600014C23 gene	46043790–46044770	2
Abcc10	224814	ABCC10	ATP-binding cassette, sub-family C (CFTR/MRP), member 10	46614147–46639278	1
Gm5093	328825		predicted gene 5093	46750504–46751023	1
Cul9	78309	CUL9/PARC	cullin 9	46811498–46857314	5
Pex6	224824	PEX6	peroxisomal biogenesis factor 6	47022389–47036467	2
Cnpy3	72029	CNPY3	canopy FGF signaling regulator 3	47046631–47063140	1
Rpl7l1	66229	RPL7L1	ribosomal protein L7-like 1	47084833–47093598	1
Gm16494	105246356		non-histone chromosomal protein HMG-17 pseudogene	47327623–47327881	2
Gm4945	240110		ribosomal protein L29 pseudogene	47353513–47353965	3
AI661453	224833		expressed sequence AI661453	47747540–47781563	3
Ccnd3	12445	CCND3	cyclin D3	47815976–47910616	1
Usp49	224836	USP49	ubiquitin specific peptidase 49	47941615–47997663	1
Prickle4	381104	PRICKLE4	prickle planar cell polarity protein 4	47999442–48005661	2
Frs3	107971	FRS3	fibroblast growth factor receptor substrate 3	47999955–48015211	1
Foxp4	74123	FOXP4	forkhead box P4	48178058–48235570	1
9830107B12Rik	328829		RIKEN cDNA 9830107B12 gene	48436215–48453439	7
A530064D06Rik	328830		RIKEN cDNA A530064D06 gene	48456296–48474443	3
Trem3	58218		triggering receptor expressed on myeloid cells 3	48554805–48565869	1
Treml1	71326	TREML1	triggering receptor expressed on myeloid cells-like 1	48666944–48674204	1
Pp2d1	110332	PP2D1	protein phosphatase 2C-like domain containing 1	53814488–53846479	1
Sult1c2	69083	SULT1C2	sulfotransferase family, cytosolic, 1C, member 2	54136665–54153367	1
Vmn2r118	383258		vomeronasal 2, receptor 118	55897370–55932192	4
Fsd1	240121	FSD1	fibronectin type 3 and SPRY domain-containing protein	56293509–56303881	1
Ticam1	106759	TICAM1	toll-like receptor adaptor molecule 1	56576319–56583786	2
Safb	224903	SAFB	scaffold attachment factor B	56891825–56913294	1
Catsperd	106757	CATSPERD	cation channel sperm associated auxiliary subunit delta	56935143–56971456	1
Arhgap28	268970	ARHGAP28	Rho GTPase activating protein 28	68149708–68311115	3
Gm16519	546695		ribosomal protein L12 pseudogene	71236053–71236541	2
Alk	11682	ALK	anaplastic lymphoma kinase	72175967–72911622	1
Vit	74199	VIT	Vitrin	78815493–78934837	1
Ndufaf7	73694	NDUFAF7	NADH: ubiquinone oxidoreductase complex assembly factor 7	79244565–79255481	1
Thada	240174	THADA	thyroid adenoma associated	84497504–84773633	1
Lrpprc	72416	LRPPRC	leucine-rich PPR-motif containing	85012675–85098217	1
Tmem247	78469	TMEM247	transmembrane protein 247	87224776–87229802	1
Mcfd2	193813	MCFD2	multiple coagulation factor deficiency 2	87561871–87573363	1
Gm10184	100043906		karyopherin (importin) alpha 2 pseudogenes	90215890–90217877	1

The distal segment of the QTL that does not overlap with the survival QTL in SOD1 transgenic mice contains 29 genes with non-synonymous variations, nine of them demonstrated variations predicted to be deleterious and/or damaging with LRPPRC as the most likely candidate genetic modifier of ALS. The leucine-rich pentatricopeptide repeat containing (LRPPRC) protein regulates mitochondrial mRNA stability and an amino-acid substitution of this protein causes the French-Canadian type of Leigh syndrome (LSFC), a progressive neurodegenerative disorder characterized by mitochondrial complex IV deficiency [27, 28].

The proximal segment that overlaps with the survival QTL in SOD1 transgenic mice contains 222 genes with non-synonymous variations, eighty-seven of them demonstrated variations predicted to be deleterious and/or damaging (Table 5). Two genes in this segment, NOTCH3 and Safb/SAFB1, have been associated with motor neuron disease. Mutations in NOTCH3 have been shown to cause cerebral autosomal dominant arteriopathy with subcortical infarcts and leukoencephalopathy (CADASIL), the most common form of hereditary stroke [29]. NOTCH3 mutations have also been associated with a number of neurodegenerative diseases such as; Alzheimer’s disease, Parkinson’s disease, multiple sclerosis, ALS and fronto-temporal lobar degeneration [29–31]. SAFB1 is an RNA binding protein implicated in the regulation of transcription, stress response, DNA repair and RNA processing [32]. A recent study suggests that SAFB1 tethers FUS to the chromatin compartment thorough N-terminal DNA-binding motif, indicating that SAFB1 could be a FUS’s functional platform in the chromatin compartment regulating RNA splicing and ligand-dependent transcription. This interaction may shed light on the etiological significance of nuclear matrix-associated proteins in ALS pathogenesis [33].

This region also contains the mouse major histocompatibility complex (H2) [34, 35]. Twenty-two genes within H2 demonstrated non-synonymous variations deemed to be deleterious and/or damaging, with some regions such as H2-K1, H2-d1, H2-Q2 and H2-Q4 having 20 or more variations each (Table 5). The Major Histocompatibility Complex has been shown to have a protective role in ALS [36, 37].

In addition, there are several genes in this segment whose function, when altered, can affect motor neuron survival such as; Marchf2, Ndufa7, Vps52, Tap1, Nelfe, Ehmt2, Vars2, Mdc1, Adgrf1, Aars2, Hsp90ab1, Abcc10, Cul9/Parc, Usp49, Foxp4, Trem3, Treml1 and Ticam1/TRIF.

MARCHF2 (MARCH-II) is a likely regulator of trafficking between the trans-Golgi network and endosomes [38] and may also function as a molecular bridge with ubiquitin ligase activity [39]. NDUFA7 is a subunit of mitochondrial complex I [40]. Genetic variants of Complex I genes may influence the nature of tissue response to inflammation in the central nervous system [41]. Vps52 is a subunit of the GARP and EARP complexes that has been shown to be involved in the regulation of neurite outgrowth [42]. The transporter associated with antigen processing 1 (TAP1) gene is involved in the pumping of degraded cytosolic peptides across the endoplasmic reticulum into the membrane-bound compartment where class I molecules assemble. The TAP1 and TAP2 proteins form the heterodimer transporter associated with antigen processing (TAP) complex Loss of TAP function leads to a loss of cell surface expression of MHC class I molecules [43]. NELFE is an essential component of the negative elongation factor (NELF) complex, which allows cells to coordinate and appropriately respond to signals by modulating the rate of transcriptional pause release [44]. Euchromatic histone-lysine N-methyltransferase 2 (EHMT2) along with EHMT1 comprises a histone methyltransferase complex (GLP/G9a) that methylates lysine residues of histone H3. This methylation is associated with gene silencing in euchromatin [45].

EHMT2 has been shown to be an epigenetic regulator of VEGFA alternative splicing [46]. VEGF has been shown to be a modifier of motoneuron degeneration both in human ALS and the G93A-hSOD1 mouse model of ALS [47]. The VARS2 nuclear gene encodes mitochondrial valyl-tRNA (Val-tRNA) synthetase. Mutations in this gene cause combined oxidative phosphorylation deficiency-20 [48] and are also associated with early-onset mitochondrial encephalopathies [49]. The MDC1 protein is part of the DNA damage response (DDR) pathway. MDC1 plays an early and important role in the DDR [50, 51]. ADGRF1 (GPR110) has been shown to regulate immune function in both brain and periphery [52]. The mitochondrial alanyl-transfer (t)RNA synthetase 2 (AARS2) has been linked to leukoencephalopathy [53]. The heat shock protein (Hsp) HSP90 is involved in protein folding, refolding, transport as well as protein degradation [54]. ABCC10 is a member of the superfamily of ATP-binding cassette (ABC) transporters, expressed in the blood brain barrier, whose function is to transport molecules across extra- and intra-cellular membranes [55]. PARC regulates p53 subcellular localization and apoptosis [56] and mediates the degradation of cytochrome c promoting neuronal survival [57]. USP49 is a histone H2B-specific deubiquitinase and has a critical role for H2B deubiquitination in cotranscriptional pre-mRNA processing events [58]. FOXP transcription factors are a protein subfamily known to coordinate the development of several organs including the Central Nervous System [59, 60]. These transcription factors, including FOXP4, are associated with neurodevelopmental disorders [61].

Variants within the TREM gene cluster are associated with the risk of developing Alzheimer’s disease, making them a therapeutic target for the treatment of neurodegenerative diseases [62, 63]. TICAM1/TRIF encodes an adaptor protein containing a Toll/interleukin-1 receptor (TIR) homology domain. TIR-domain-containing adapter-inducing interferon-β (TRIF) is an adapter in responding to activation of toll-like receptors. Mutations in this gene are associated with acute infection-induced encephalopathies. TRIF is dynamically modified by ubiquitination and deubiquitination, which plays a critical role in regulating its activity. Toll/IL-1R domain-containing adaptor-inducing IFN-β (TRIF)-dependent signaling is required for TLR-mediated production of type-I IFN and several other proinflammatory mediators. The TRIF pathway contributes to control of both viral and bacterial pathogens through promotion of inflammatory mediators and activation of antimicrobial responses [64].

The QTL on Chr 18 contains 85 genes were SJL mice demonstrate non-synonymous variations (S6 Table). Thirty of those genes have PROVEAN or SIFT scores that predict the variations to be deleterious and/or damaging to protein function (Table 6).

**Table 6:** Protein coding genes in the Chr18 QTL of G59S-hDCTN1 mice demonstrating non-synonymous variations deemed to be deleterious and/or damaging.

Mouse Gene Symbol	Mouse Gene ID	Human Gene Symbol	Mouse Gene Name	Chr 18 Location GRCm39	Del/Dam # Vars
Gm10036	100040551		ribosomal protein L11 pseudogene	15965851–15966384	1
Gm10549	433171		predicted gene 10549	33597216–33607763	1
Brd8	78656	BRD8	bromodomain containing 8	34731668–34757654	1
Kdm3b	277250	KDM3B	KDM3B lysine (K)-specific demethylase 3B	34910100–34971713	1
Egr1	13653	EGR1	early growth response 1	34992876–34998037	1
Hspa9	15526	HSPA9	heat shock protein 9	35070467–35087410	1
Matr3	17184	MATR3	matrin 3	35695191–35726888	5
Wdr55	67936	WDR55	WD repeat domain 55	36893273–36896863	1
Pcdha1	116731	PCDHA1	protocadherin alpha 1	37063237–37320714	2
Pcdhb2	93873	PCDHB2	protocadherin beta 2	37427865–37430677	3
Pcdhb3	93874	PCDHB3	protocadherin beta 3	37433852–37437638	4
Pcdhb4	93875	PCDHB4	protocadherin beta 4	37440508–37444225	5
Pcdhb5	93876	PCDHB5	protocadherin beta 5	37453434–37456968	7
Pcdhb6	93877	PCDHB6	protocadherin beta 6	37466974–37470727	3
Pcdhb8	93879	PCDHB8	protocadherin beta 8	37488174–37491657	9
Pcdhb9	93880	PCDHB9	protocadherin beta 9	37533908–37536962	12
Diaph1	13367	DIAPH1	diaphanous related formin 1	37976654–38068529	1
Pcdh12	53601	PCDH12	protocadherin 12	37568674–37571707	1
Spink14	433178	SPINK14	serine peptidase inhibitor, Kazal type 14	44160936–44165484	1
Dtwd2	68857	DTWD2	DTW domain containing 2	49829212–49888668	3
Srfbp1	67222	SRFBP1	serum response factor binding protein 1	52598765–52625003	1
Fbn2	14119	FBN2	fibrillin 2	58141695–58343559	1
Cdx1	12590	CDX1	caudal type homeobox 1	61151934–61169271	1
Arhgef37	328967	ARHGEF37	Rho guanine nucleotide exchange factor (GEF) 37	61624728–61669665	1
Gm9949	225609		predicted gene 9949	62313197–62317476	3
Alpk2	225638	ALPK2	alpha-kinase 2	65398600–65527137	2
Oacyl	319888		O-acyltransferase like	65831339–65884672	1
Ldlrad4	52662	LDLRAD4	low density lipoprotein receptor class A domain containing 4	68066328–68401701	1
Rnmt	67897	RNMT	RNA (guanine-7-) methyltransferase	68433426–68457923	2
Poli	26447	POLI	polymerase (DNA directed), iota	70641751–70663691	1

MATR3 and the protocadherin family of genes are the most likely ALS genetic modifying candidates in this QTL. MATR3 is the one gene in the Chr 18 QTL that has been directly linked to ALS [65, 66]. Mutations in the RNA/DNA-binding protein MATR3, which is known to interacts with TDP-43, has been shown to cause ALS [67].

There are 14 genes in the protocadherin family demonstrating non-synonymous variations in SJL/J mice within the Chr 18 QTL. Protocadherins are the largest mammalian subgroup of the cadherin superfamily [68]. Preventing the interaction of axons and dendrites from the same neuron during development, is mediated through the stochastic single-neuron expression of clustered protocadherin protein isoforms [69]. Most of the protocadherin proteins demonstrating variations belong to the alpha and beta clusters. The one gene in the gamma cluster (Pcdhgc3) demonstrated one non-synonymous SNP resulting in a single amino acid change (S651N) that was predicted by PROVEAN to be neutral, tolerated. Nine genes in the protocadherin family; Pcdha1, Pcdhb2, Pcdhb3, Pcdhb4, Pcdhb5, Pcdhb6, Pcdhb8, Pcdhb9 and Pcdh12 demonstrated non-synonymous variations with PROVEAN or SIFT scores that predict the variations to be deleterious and/or damaging to protein function. Protocadherins within the alpha cluster have been shown to contribute to neural circuit development [70]. Protocadherins in the beta cluster contribute to specifying the identity and diversity of individual neurons [71] and members of this cluster have been reported to control axon growth in zebrafish motor neurons [72].

In addition, there are several genes in this QTL demonstrating variations predicted to be deleterious and/or damaging, whose function, when altered, can affect motor neuron survival such as; Brd8, Kdm3b, Egr1, Hsp9 and Diaph1. BRD8 (p120) has been linked with DNA repair [73–75]. KDM3B has been identified as an antioxidant gene [76]. EGR1 is a transcription factor also known as nerve growth factor-induced protein A (NGFIA). EGR1 transcriptional regulation is complex and involves; transcription factor binding, cofactors recruitment, chromatin dynamics including histone methylation, acetylation and phosphorylation, as well as nucleosome positioning [77]. HSPA9 (Mortalin) is a mitochondrial chaperone of the heat shock protein 70 family [78, 79]. Mutations in HSPA9 may contribute to the risk of developing Parkinson’s disease [80]. Mutations in DIAPH1 results in mitochondrial dysfunction and immunodeficiency which can lead to seizures, cortical blindness, hearing loss and microcephaly syndrome [81, 82].

Our results shows that mice with SJL genotype at the Chr17 QTL demonstrated shorter survival than mice with C57BL6 genotype, whereas at the Chr18 QTL mice with the SJL genotype demonstrated longer survival than mice with the C57BL6 genotype. The phenotypic changes can result from alterations in protein coding genes within the QTLs, however they could also arise from altered non-coding RNAs such as microRNAs or long non-coding RNAs.

In addition, altered survival does not necessarily mean that all SJL-derived protein coding genes and/or non-coding RNAs at the Chr 17 QTL are deleterious or all beneficial at the Chr 18 QTL. It means that the total contribution of genetic elements that differ in SJL mice at the Chr 17 QTL results in reduced survival, whereas the total contribution of those at the Chr 18 QTL promotes increased survival. We hypothesize that the variations in survival demonstrated by mice with SJL alleles within the Chr 17 and 18 QTLs are due to either allelic non-synonymous genetic polymorphism(s) of protein coding genes and/or non-coding RNAs or their differential expression.

We further hypothesize that some of the genetic modifiers located in the Chr 17 region where the SOD1 and dynactin-1 QTLs overlap, will be shared by these two MND models despite the fact that motor neuron degeneration is caused by mutations in different proteins. We are currently undertaking studies using interval specific congenic (ISC) mice on a C57BL6/J background with Chr 17 SJL intervals in the region where the SOD1 and dynactin-1 QTLs overlap. We have bred the SOD1 and dynactin-1 transgenes into these ISC lines in order to determine which regions modify the survival of these mice. Our goal is to generate ISC mice with 1–3 Mb intervals that preserve the altered phenotype. These mice can then be used for the identification of genetic modifiers of motor neuron disease. The identification of genetic modifiers of motor neuron disease, especially those modifiers that are shared by SOD1 and dynactin-1 transgenic mice, may help to develop biomarkers predictive of disease progression. In addition, they will identify pathways that are important in motor neuron degeneration and lead to novel targets for therapies that can alter the course of this devastating illness.

## Supporting information

S1 Dataset Data dynactin F2 mice phenotype and genotype. (XLSX)

S1 Fig Effect plot of genotype on health sacrifice at the chromosome 6 QTL. Effect of genotype at chromosome 6, 33.5 cM on health sacrifice. The health sacrifice in days is plotted as the mean + SE. The genotypes are BB for homozygous B6, SS for homozygous SJL and BS for heterozygous B6/SJL.
(TIF)

S2 Fig Effect plot of genotype on health sacrifice at the chromosome 15 QTL. Effect of genotype at chromosome 15, 16.3 cM on health sacrifice. The health sacrifice in days is plotted as the mean + SE. The genotypes are BB for homozygous B6, SS for homozygous SJL and BS for heterozygous B6/SJL.
(TIF)

S1 Table Single nucleotide polymorphisms across the mouse genome used to differentiate C57BL/6J from SJL/J mice. (XLSX)

S2 Table One-dimensional scans for the survival phenotype with pval less than or equal to 0.63. (XLSX)

S3 Table One-dimensional scans for the health sacrifice phenotype with pval less than or equal to 0.63. (XLSX)

S4 Table Two-dimensional scans for the survival and health sacrifice phenotypes with pvals less than or equal to 0.63. (XLSX)

S5 Table Gene locations in the chromosome 17 QTL where SJL mice demonstrate non-synonymous variations. (XLSX)

S6 Table Gene locations in the chromosome 18 QTL where SJL mice demonstrate non-synonymous variations. (XLSX)
