# Supplementary figures and images for: Identification of quantitative trait loci for survival in the mutant dynactin p150Glued mouse model of motor neuron disease
Source: PLoS One. 2022 Sep 15;17(9):e0274615. doi: 10.1371/journal.pone.0274615 (PMC9477371; doi:10.1371/journal.pone.0274615)

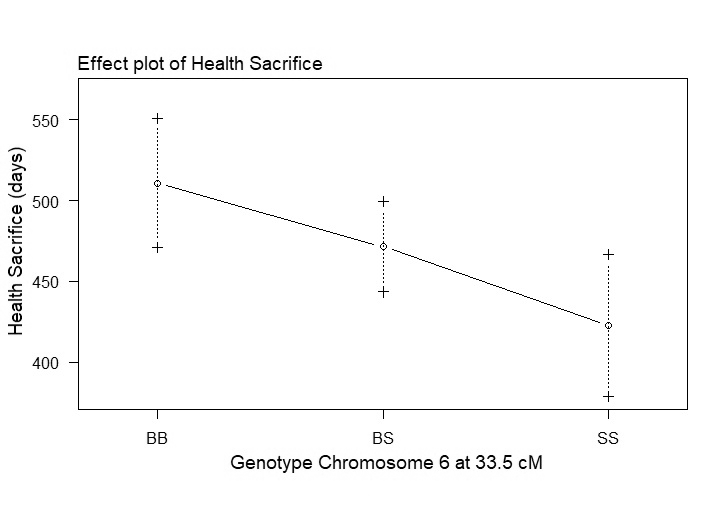

Supplement: S1 Fig — Effect of genotype at chromosome 6, 33.5 cM on health sacrifice. The health sacrifice in days is plotted as the mean + SE. The genotypes are BB for homozygous B6, SS for homozygous SJL and BS for heterozygous B6/SJL. (TIF) [file pone.0274615.s002.tif]

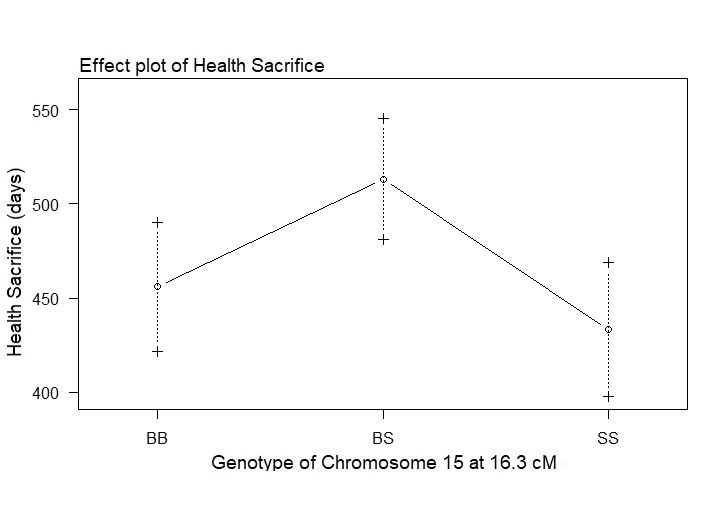

Supplement: S2 Fig — Effect of genotype at chromosome 15, 16.3 cM on health sacrifice. The health sacrifice in days is plotted as the mean + SE. The genotypes are BB for homozygous B6, SS for homozygous SJL and BS for heterozygous B6/SJL. (TIF) [file pone.0274615.s003.tif]
